# Opening pressures and intratidal opening and closing in ARDS lung

**DOI:** 10.1186/cc13484

**Published:** 2014-03-17

**Authors:** I Algieri, D Chiumello, M Cressoni, A Colombo, G Babini, S Luoni, M Brioni, F Crimella, C Chiurazzi, B Cormini, M Monti

**Affiliations:** 1 Universitá degli Studi di Milano, Milan, Italy; 2 Fondazione IRCCS Cá Granda Ospedale Maggiore Policlinico, Milan, Italy

## Introduction

Intratidal opening/closing is believed to be one of the main triggers of ventilator-induced lung injury; the application of higher PEEP united with the limitation of plateau pressure at 30 cmH_2_O may limit this phenomenon. We aim to evaluate the intratidal opening/ closing at different PEEP levels and the amount of lung parenchyma that regains inflation going from 30 (pressure limit of tidal ventilation) to 45 cmH_2_O.

## Methods

Patients with ARDS underwent whole-lung low-dose (60 mAs, 120 kV) computed tomography at PEEP 5 cmH_2_O end expiration and end inspiration, PEEP 15 cmH_2_O end expiration and end inspiration, and airway pressure 30 and 45 cmH_2_O end inspiration. Quantitative analysis of CT data was performed and recruitability was defined as the fraction of lung parenchyma that regains inflation going from 5 cmH_2_O end expiration to 45 cmH_2_O end inspiration. Patients were classified as high recruiters (HR) and low recruiters (LR) according to the median lung recruitability (14% of lung parenchyma).

## Results

Eighteen patients (male 12, age 56.0 ± 18.6 years, BMI 25.6 ± 5.2 kg/m^2^, PaO_2_/FiO_2 _166 ± 70, tidal volume 496 ± 84 ml, RR 19 ± 7.4 breaths/minute, PEEP 11.4 ± 3.7 cmH_2_O) were enrolled. The fraction of lung parenchyma that could be recruited at plateau pressure above 30 cmH_2_O was highly variable with a median of 5% (IQ range 1 to 17%) corresponding to a median 16% (IQ range 10 to 47%) of total lung recruitability. Indeed we observed a statistically relevant difference in lung recruitment between airway pressures of 30 and 45 cmH_2_O (*P *= 0.016). With PEEP 5 cmH_2_O, median opening/closing was 129 g (IQ range 124 to 145 g) in the HR group and 50 g (IQ range 24 to 76 g) in LR (*P *= 0. 006), corresponding to 8% of lung parenchyma (IQ range 6 to 8%) in HR and 4% (2 to 7%) in LR (*P *= 0.053). Increasing the PEEP level to 15 cmH_2_O, median opening/closing was 67 g (IQ range 24 to 95 g) in the HR group and 45 g (0 to 80 g) in the LR group (*P *= 0.512), corresponding to 3% (1 to 5%) in HR and 3% (0 to 5%) in LR (*P *= 0.93). We observed a statistical difference between recruitments with PEEP 5 and PEEP 15 in the HR group (*P *= 0.013) but not in the LR group (*P *= 0.781).

## Conclusion

A highly variable and significant fraction of lung parenchyma is always closed with tidal ventilation at 30 cmH_2_O plateau pressure, regardless of the PEEP level applied; this implies that sigh or periodic recruitment maneuvers may lead to opening and closing. Intratidal opening/closing was reduced but not abolished in all patients while ventilating at higher PEEP level (15 cmH_2_O).

